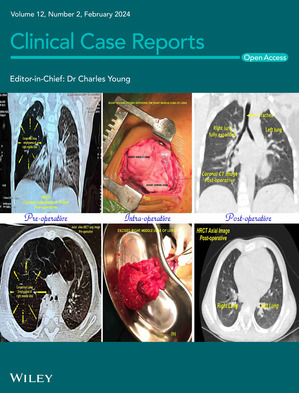# Cover Image

**DOI:** 10.1002/ccr3.8557

**Published:** 2024-03-07

**Authors:** Jigar Prabhulal Thacker, Vishal Vinayak Bhende, Tanishq Shashikant Sharma

## Abstract

The cover image is based on the Case Report *Congenital lobar emphysema: A diagnostic dilemma with coexistent congenital heart defects* by Jigar Prabhulal Thacker *et al.*, https://doi.org/10.1002/ccr3.8538